# Presence of Germline and Full-Length IgA RNA Transcripts Among Peritoneal B-1 Cells

**DOI:** 10.1155/1998/37576

**Published:** 1998

**Authors:** Rick De Waard, Peter M. Dammers, James W. Tung, Aaron B. Kantor, Jennifer A. Wilshire, Nicolaas A. Bos, Leonore A. Herzenberg, Frans G. M. Kroese

**Affiliations:** 1 Department of Histology and Cell Biology University of Groningen Groningen 9713 EZ The Netherlands; 2 Department of Genetics Stanford University Medical School Stanford California 94305 USA; 3 AmCell Corporation 1190 Bordeaux Drive Sunnyvale California 94089 USA; 4 Department of Histology and Cell Biology University of Groningen Oostersinge169-1 Groningen 9713 EZ The Netherlands

**Keywords:** B-1 cells, germline transcripts, IgA

## Abstract

Next to conventional B cells (or B-2 cells), peritoneal B-1 cells have been shown to contribute significantly to the production of IgA-secreting plasma cells in the gut. Evidence for this was mainly based on studies comprising manipulated animals, including lethally X-irradiated and transgenic mice. To examine the ability of peritoneal B-1 cells from untreated mice to switch actively to IgA *in vivo*, we performed RT-PCR analysis on FACS-sorted peritoneal B-cell subsets from untreated BALB/c mice in order to examine the presence of germline Cα mRNA and mature Cα mRNA transcripts. Germline Cα and mature Cα transcripts were readily detectable in peritoneal B-1 cells (defined as IgM^bright^/IgD^dull^), but not, or very little, in peritoneal B-2 cells (defined as IgM^dull^/IgD^bright^). Moreover, by subdividing the B-l-cell population in CD5^+^ B-1a cells and CD5^-^ B-1b cells, it was shown that *in vivo* expression of germline Cα and mature Cα transcripts was largely restricted to the B-1b-cell lineage. These results indicate that peritoneal B-1 cells indeed are capable to switch to IgA under normal physiological conditions and hereby further support the view that B-1 cells contribute significantly to the mucosal IgA response, albeit this function appears to be restricted to the B-1b-cell subset.


*Developmental Immunology*, 1998, Vol. 6, pp. 81–87
Reprints available directly from the publisher
Photocopying permitted by license only

© 1998 OPA (Overseas Publishers Association)
N.V. Published by license under the
Harwood Academic Publishers imprint,
part of the Gordon and Breach Publishing Group
Printed in Malaysia

### Presence of Germline and Full-Length IgA RNA Transcripts Among Peritoneal B-1 Cells

RICK DE WAARD[a], PETER M. DAMMERS[a], JAMES W. TUNG[b], AARON B. KANTOR[c], JENNIFER A. WILSHIRE[b], NICOLAAS A. BOS[a], LEONORE A. HERZENBERG[b] and FRANS G. M. KROESE[a*]

[a]*Department of Histology and Cell Biology, University of Groningen, 9713 EZ Groningen, The Netherlands*; [b]*Department of Genetics, Stanford University Medical School, Stanford, California 94305, USA*; [c]*AmCell Corporation, 1190 Bordeaux Drive, Sunnyvale, California 94089, USA*

(*In final form 30 May 1997*)

Next to conventional B cells (or B-2 cells), peritoneal B-1 cells have been shown to contribute significantly to the production of IgA-secreting plasma cells in the gut. Evidence for this was mainly based on studies comprising manipulated animals, including lethally X-irradiated and transgenic mice. To examine the ability of peritoneal B-1 cells from untreated mice to switch actively to IgA *in vivo*, we performed RT-PCR analysis on FACS-sorted peritoneal B-cell subsets from untreated BALB/c mice in order to examine the presence of germline $C\alpha$ mRNA and mature $C\alpha$ mRNA transcripts. Germline $C\alpha$ and mature $C\alpha$ transcripts were readily detectable in peritoneal B-1 cells (defined as $IgM^{bright}/IgD^{dull}$), but not, or very little, in peritoneal B-2 cells (defined as $IgM^{dull}/IgD^{bright}$). Moreover, by subdividing the B-1-cell population in $CD5^+$ B-1a cells and $CD5^-$ B-1b cells, it was shown that *in vivo* expression of germline $C\alpha$ and mature $C\alpha$ transcripts was largely restricted to the B-1b-cell lineage. These results indicate that peritoneal B-1 cells indeed are capable to switch to IgA under normal physiological conditions and hereby further support the view that B-1 cells contribute significantly to the mucosal IgA response, albeit this function appears to be restricted to the B-1b-cell subset.

*Keywords:* B-1 cells, germline transcripts, IgA

### INTRODUCTION

The intestinal lamina propria is characterized by a mucosal preponderance of IgA-secreting plasma cells (Van der Heijden et al., 1987). In the mouse, these cells have been shown to originate from two different lineages of B cells. An important source for IgA-secreting plasma cells is formed by Peyer's patch B cells (Craig and Cebra, 1971) of which nearly all are bone-marrow-derived conventional type B cells or B-2 cells ($IgM^{dull}$, $IgD^{bright}$). After antigen stimulation, committed B cells leave the Peyer's patches, migrate via mesenteric lymph nodes and thoracic duct into the circulation to the spleen, and finally home back to the

*Corresponding author. Present address: Department of Histology and Cell Biology, University of Groningen, Oostersingel 69-1, 9713 EZ Groningen, The Netherlands.

gut lamina propria. During the migration, the cells expand, differentiate, and mature into IgA-secreting plasma cells (Tseng, 1984).

Another source of progenitor cells that contribute to the pool of lamina propria IgA plasma cells is formed by B-1 cells (Kroese et al., 1989, 1995), which reside abundantly in the murine peritoneal cavity (Herzenberg et al., 1986). The B-1 cell population (defined as $IgM^{bright}$, $IgD^{dull}$) can be divided, based on surface CD5 expression, into a $CD5^{+}$ B-1a cell and a $CD5^{-}$ B-1b "sister" cell population (Kantor and Herzenberg, 1993). The B-1-cell lineages differ from the conventional B-cell populations with respect to cell-surface-marker expression, localization, development, and antibody repertoire (Stall et al., 1996). Conventional B cells may undergo somatic hypermutation in the germinal centers and are responsible for high-affinity antibody responses to various antigens, whereas B-1 cells, which show a very low frequency of hypermutation of their Vh genes (Kantor, 1996), primarily produce low-affinity IgM immunoglobulins, most of which are cross-reactive with bacteria-related and self-antigens (reviewed in Kroese et al., 1996).

Evidence that B-1 cells are involved in IgA production was demonstrated in different chimeric mouse models and B6-Sp6 $\mu,\kappa$ transgenic mice (reviewed in Kroese et al., 1995). Additionally, it was shown that several B-1-cell lines showed a high frequency of switching to IgA (Whitmore et al., 1991). However, all these studies comprised manipulated *in vivo* mouse models or *in vitro* experiments that left many questions regarding the B-1-cell lineage in normal untreated mice unanswered.

IgA class switching of $IgM^{+}$ B lymphocytes is preceded by the synthesis of germline $C\alpha$ mRNA transcripts (Lebman et al., 1990). Germline IgA transcription starts 5′ of an $I\alpha$ exon, proceeds through the switch region, and terminates downstream of the $C\alpha$ exons. This leads to splicing of the $I\alpha$ exon to the $C\alpha$ exon, which forms the germline IgA transcript (Lebman et al., 1990). Downregulation of germline IgA transcripts has been shown to inhibit IgA isotype switching that implies a role for IgA germline transcripts prior to the expression of full-length IgA transcripts (Lin and Stavnezer, 1992).

To study the ability of peritoneal B-1 cells (both B-1a and B-1b) to switch to IgA under normal physiological conditions, we sorted peritoneal B-cell subpopulations from untreated BALB/c mice and examined those populations for the presence of germline IgA transcripts and full-length IgA mRNA transcripts.

### RESULTS AND DISCUSSION

### Peritoneal Washout Cells Express Germline $C\alpha$ Transcripts

The presence of germline $C\alpha$ mRNA transcripts and mature IgA heavy-chain mRNA transcripts was determined by RT-PCR in unsorted peritoneal cells from 3-month-old untreated BALB/c mice. cDNA was synthesized from mRNA extractions of peritoneal washout cells as well from unsorted Peyer's patch cells, which already have been shown to express $C\alpha$ and mature IgA transcripts (Weinstein et al., 1991). Sorted splenic T cells served as negative controls. Additionally, all cell suspensions were tested for mature $C\mu$ transcripts. The $C\alpha$ germline transcripts were revealed by the use of the $I\alpha$-leader and $C\alpha1$-$C\alpha2$ primers, whereas full-length IgA and IgM transcripts were identified by usage of an universal Vh primer in combination with $C\alpha$ or $C\mu$ primers, respectively. In each set of experiments, cDNAs of various different cell suspensions were normalized by means of serial dilutions with $\beta$-actin mRNA (650 bp). The specificity of the primers was tested on RNA derived from mouse IgA and IgM secreting hybridoma cell lines 2F7 (Bos et al., 1996) and NEO4211 (Bos and Meeuwsen, 1989), respectively. The germline primer did not result in a PCR product in either cell lines, whereas the universal Vh primer in combination with the $C\alpha$ primer and $C\mu$ primer did result in the correct PCR product for the corresponding hybridoma (Table I). Both unsorted peritoneal cells and Peyer's patches cells express

TABLE I Expression of Germline IgA Transcripts, Full-Length IgA and IgM mRNA, and β-Actin

| | Per C | PP | T cells | B-2 | B-1 | B-1a | B-1b | 2F7 | NEO4211 |
|---|---|---|---|---|---|---|---|---|---|
| β-actin | + | + | + | + | + | + | + | + | + |
| Cα-GLT | + | + | − | − | + | − | + | − | − |
| IgM | + | + | − | + | + | + | + | − | + |
| IgA | + | + | − | − | + | − | + | + | − |

Note: + denotes mRNA levels detectable after 40 cycles of RT-PCR using cDNA from the different sorted and unsorted lymphocyte populations.

germline Cα RNA (445 bp) and mature Cα transcripts (490 bp) (Table I).

### Germline IgA Transcript Expression Is Restricted To Peritoneal B-1 Cells

To specify the phenotype of peritoneal B cells that switch to IgA, peritoneal B-cell subpopulations were sorted based on differences in IgM and IgD expression. The $IgM^{bright}$ and $IgD^{dull}$ populations are formed by the B-1 cells, whereas the B-2 cells are found within the $IgM^{dull}$ and $IgD^{bright}$ populations. Figure 1 shows a typical example of the staining used and the sorting gates set. The purity of the two sorted peritoneal B-cell subsets was approximately 95%. RNA was extracted and cDNA was synthesized from the sorted B-cell fractions and analyzed for mature IgM, mature IgA, and germline Cα transcripts.

Cα germline transcript and mature IgA expression occurred mainly in the peritoneal B-1-cell subpopulation (Figure 2 and Table I). In some B-2-cell sorts, a 20-50-fold lower expression of Cα germline transcript and/or mature IgA transcripts compared to the B-1-cell sorts could be detected. This could be due to contaminating B-1 cells, since in these sorts higher percentages of B-1-cell contamination was observed.

Our parameter for determining switching to IgA is the expression of germline Cα and full-length IgA mRNA transcripts. Germline transcripts are translated preceding the actual isotype switching. A strong correlation between germline transcript expression and isotype switching has been shown (Lebman et al., 1990). However, the function of germline transcripts is less clear, since mice lacking the Iα exon are still able to switch to IgA (Harriman et al., 1996).

Our data do not provide information on the proportion of B1 cells expressing germline or mature IgA transcripts. Previously, we have found by FACS analysis only a very low surface IgA expression on peritoneal B-1 cells (<1% $IgA^+$ B-1 cells) (Kroese et al., 1989). The mouse peritoneal cavity might thus provide the required microenvironment for B-1 cells to initiate switching to IgA *in vivo*, but most likely

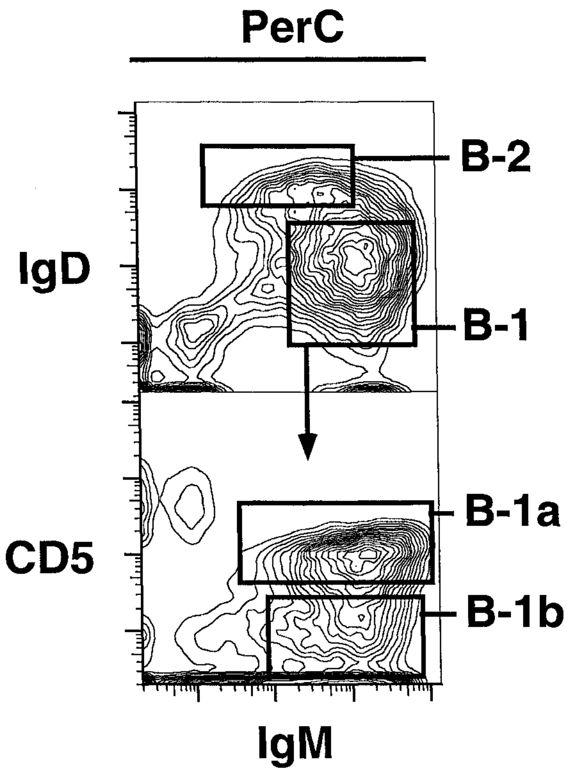

FIGURE 1 Identification and sorting of peritoneal B-cell subpopulations by flow cytometry. Peritoneal washout cells of 3-month-old untreated BALB/c mice were stained with anti-IgM (FL) [331], anti-IgD (PE) [11-26], and anti-CD5 (APC) [53-78]. Shown gates for sorting were set on the basis of IgM, IgD, and CD5 expression.

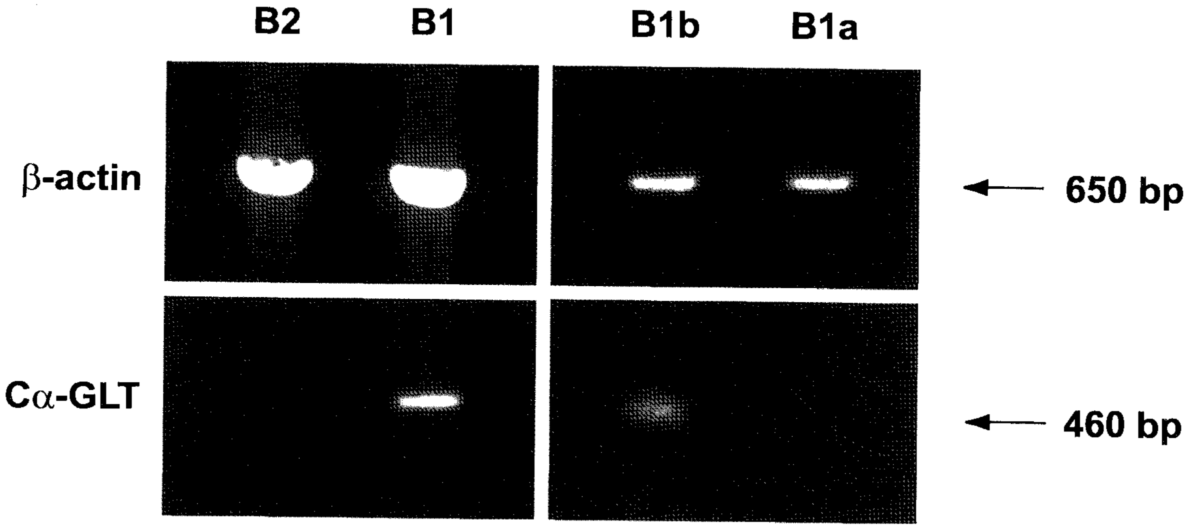

FIGURE 2 Germline Cα transcript expression in sorted peritoneal B-cell subpopulations. Expression of germline Cα transcripts was determined by RT-PCR analysis of RNA from 2 × $10^5$ sorted B cells. The amount of cDNA of the different B-cell populations used in the PCR reactions were equalized by comparing with β-actin PCR products derived from serial diluted cDNAs. The size of the expected PCR products for β-actin and germline Cα transcripts RNA are 650 and 445 bp, respectively.

they differentiate and mature to IgA plasma cells after migration out of the peritoneal cavity. For Peyer's patch B cells, it is known that they leave those sites after initiation of isotype switching and migrate through the circulation toward the lamina propria while they further differentiate to IgA plasma cells (Tseng, 1984). Where and how B-1 cells migrate from the peritoneal cavity and differentiate to IgA plasma cells in the lamina propria remain to be established.

Also the factors that are involved in the initiation of isotype switching of B-1 cells in the peritoneal cavity are still unknown. Different interleukins, such as IL-4, IL-5, and TGF-β, already have been shown to play a role in the regulation of IgA switching and secretion (Harriman et al., 1988; Lebman and Coffman, 1988). Additionally, CD40 ligation by direct B-cell-T-cell interaction seems to be important for isotype switching (Jumper et al., 1994). Both peritoneal T cells as well as other cell types, such as mesothelial cells, might produce the correct cytokines and provide the stimuli for B-1 cells to start isotype switching to IgA. Whether peritoneal B-1 cells also can switch to other isotypes such as IgG and IgE remains also to be established.

### IgA Expression Within the Peritoneal Cavity Is Confined to the B-1b-Cell Population

The B-1-cell population consists both of $CD5^+$ B-1a cells and $CD5^-$ B-1b cells. To examine the ability of both peritoneal B-1a- and B-1b-cell subpopulations to switch to IgA, the subsets were sorted (Figure 1), RNA was isolated and cDNA was synthesized. Both B-1a and B-1b cells express mature IgM transcripts, however, the expression of germline Cα and mature IgA transcripts appeared to be largely confined to the peritoneal B-1b cells (Table I and Figure 2). The PCR products of the B-1b-cell population has also been cloned and sequenced, which confirmed the identity of the product as germline Cα transcripts compared to the EMBL sequence databank (data not shown). In one out of four experiments also, a very low expression of germline Cα transcripts was found in the B-1a subset, which was most probably due to contaminating B-1b cells in the B-1a-cell fraction.

Two alternative explanations can explain this preferential expression of IgA transcripts among B-1b cells. First, it might be argued that B-1a cells downregulate CD5 expression after switching to IgA. This might explain reconstitution experiments with purified B-1a cells, which resulted in IgA plasma cells in the lamina propria (Beagley et al., 1995) and our own preliminary experiments with sorted peritoneal B1 cells showing that both B-1a and B-1b cells may contribute to the pool of intestinal IgA plasma cells (Kroese et al., unpublished observations). Alternatively, B-1a and B-1b cells might belong to closely related, but separate B cell lineages, whereby the switching to IgA is mainly restricted to the B-1b-cell lineage.

In conclusion, our experiments show that peritoneal B1 cells actively switch toward IgA *in vivo*, thereby confirming our previous data in manipulated animals that B-1 cells contribute to the IgA production in the mouse. The function of B-1-cell-derived IgA might be important in the establishment and maintenance of the normal gut flora, since we have shown that B-1-cell-derived monoclonal IgA antibodies primarily react with normal gut bacteria (Bos et al., 1996).

### MATERIAL AND METHODS

### Animals

Both male and female BALB/c mice at 3 to 4 months were studied. Mice were bred in the animal facility at the Stanford Department of Genetics.

### Antibodies

Rat and mouse monoclonal antibodies used in this study were as follows: Rat anti-mouse IgD (11-26), rat anti-mouse IgM (331), rat anti-mouse Ly-1 (CD5; 53-78), mouse anti-mouse Igh-6a (IgM of "a" allotype, DS-1), and mouse anti-mouse Igh-5a (IgD of "a" allotype, AMS 9.1). Purification and conjugation of antibodies to biotin, fluorescein, phycoerythrin, and allophycocyanin (APC) are described elsewhere (Hardy et al., 1984).

### Cell Preparation

Peritoneal washout cells were obtained from the mouse peritoneal cavity by injection of chilled deficient RMPI 1640 medium (Irvine Scientific, Santa Ana, CA) supplemented with 10 mM Hepes, 3% newborn calf serum and 0.1% $NaN_3$. Single-cell suspensions from spleen and Peyer's patches were prepared by mincing tissue fragments in the same medium between the frosted ends of micoscope slides. All cell suspensions were treated with red-blood-cell lysing buffer to eliminate erythrocytes.

### Staining and Cell Sorting

The B-cell subpopulations were sorted on an extensively modified fluoresence-activated cell sorter (FACS II; Becton-Dickinson, Mountain View, CA), as described (Hardy et al., 1984). For sorting subpopulations, single-cell suspensions were stained in tubes on ice with optimal concentrations of conjugated antibodies. Biotinylated antibodies were detected with avidin-Texas Red. Dead cells were stained with propidium iodide and were excluded from sorting. After sorting, 30,000 viable sorted cells were reanalyzed to test the purity of the sorted cells.

### RT-PCR

Total RNA, isolated from different cell suspensions by use of TRIzol (Life Technologies) according to the manufacturer' instructions, was used as a template for cDNA synthesis in a 30-$\mu$l reaction mix containing 1.6 $\mu$g oligo-dT 12-18 (Pharmacia), 10 mM dNTP mix (Pharmacia), MilliQ-DEPC, First Strand Buffer (Life Technologies), 0.1 mM DTT (Life Technologies), 30 U RNA quard (Pharmacia), and 200 U Superscript™ II (Life Technologies). The reaction proceeded at 45°C for 30 min, followed by heating to 94°C for 5 min. For the RT-PCR, an aliquot of cDNA

was added to a cocktail composed of 5 $\mu$l (10×) RT-PCR buffer (Life Technologies), 0.5 $\mu$l 20 mM dNTP mixture, 1.5 $\mu$l MgCl2 (Life Technologies), 2.5 $\mu$l 1% W-1 (Life Technologies), 0.6 $\mu$l 5′ primer (25-35 pmol/$\mu$l), 0.6 $\mu$l 3′ primer (25-35 pmol/$\mu$l), and 0.5 $\mu$l Taq DNA polymerase (5 U/ml) (Life Technologies). Primers sequences, 5′ to 3′, used in this study were as follows:

3′ $\beta$-actin: TCTTCATGGT GCTAGGAGCCA
5′ $\beta$-actin: CCTAAGGCCAACCGTGAAAAG
I$\alpha$-leader: CCAGTCCTAAGCTTTCTACCATAG
C$\alpha$1-C$\alpha$2: GAGGAGTAGGACCAGAGCAATTC (Bos et al., 1996)
C$\alpha$1-BamHI: CTCGGATCCTCACATT CATCGTGCC (Bos et al., 1996)
Universal Vh: ACGAATTCAGGTSMARCTG-CAGSAGTCWGG (M = A or C; R = A or G; S = C or G; W = A or T) (Orlandi et al., 1989)
C$\mu$ 1.3: CCCTGGATGACTTCAGTGTTG

The RT-PCR was performed on a thermal cycler (Pharmacia LKB—Gene ATAG Controller) with an initial denaturation cyclus at 94°C (2 min), an annealing step at 60°C (1 min), and an extension step at 72°C (1 min), respectively. Then 39 steps of amplification were performed (each step of the cycle: 1 min) with a denaturing temperature of 94°C, an annealing temperature of 60°C, and an extension temperature of 72°C. The PCR products (10 $\mu$l per reaction) were analyzed on an 1.2% ethidium-bromide-agarose gel and visualized in UV light.

***Acknowledgements***

This work was partly supported by NATO Grant CRC 910195 to Frans G.M. Kroese and Leonore A. Herzenberg and by a grant of the Interuniversity Institute for Radiopathology and Radioprotection (IRS).

The authors wish to thank Kathy Seidl and Rachel Gerstein for their technical assistance.

***References***

Beagley K.W., Murray A.M., McGhee J.R. and Eldridge J.H. (1995). Peritoneal cavity CD5 (B1a) B cells: Cytokine induced IgA secretion and homing to intestinal lamina propria in SCID mice. Immunol. Cell. Biol. **73**: 425.

Bos N.A., Bun J.C.A.M., Popma S.H., Cebra E.R., Deenen G.J., Cammen M.J.F. v.d., Kroese F.G.M., and Cebra J.J. (1996). Monoclonal Immunoglobulin A derived from peritoneal B cells is encoded by both germ line and somatically mutated VH genes and reactive with commensal bacteria. Inf. and Imm. **64**: 616-623.

Bos N.A., and Meeuwsen C.G. (1989). B cell repertoire in adult antigen-free and conventional neonatal BALB/c mice. I. Preferential utilization of the $C_H$-proximal $V_H$ gene family PC7183. Eur. J. Immunol. **19**: 1811-1815.

Craig S.W., and Cebra J.J. (1971). Peyer's patches: An enriched source of precursors for IgA-producing immunocytes in the rabbit. J. Exp. Med. **134**: 188-200.

Hardy R.R., Hayakawa K., Parks D.R., and Herzenberg L.A. (1984). Murine B cell differentiation lineages. J. Exp. Med. **159**: 1169-1188.

Harriman G.R., Bradley A., Das S., Rogers-Fani P., and Davis A.C. (1996). IgA class switch in I$\alpha$ exon-deficient mice. Role of germline transcription in class switch recombination. J. Clin. Invest. **97**: 477-485.

Harriman G.R., Kunimoto D.Y., Elliott J.F., Paetkau V., and Strober W. (1988). The role of IL-5 in IgA B cell differentiation. J. Immunol. **140**: 3033-3039.

Herzenberg L.A., Stall A.M., Lalor P.A., Sidman C., Moore W.A., Parks D.R., and Herzenberg L.A. (1986). The Ly-1 B cell lineage. Immunol. Rev. **93**: 81-102.

Jumper M.D., Splawski J.B., Lipsky P.E., and Meek K. (1994). Ligation of CD40 induce sterile transcripts of multiple Ig H chain isotypes in human B cells. J. Immunol. **152**: 438-445.

Kantor A.B. (1996). V-gene usage and N-region insertions in B-1a, B-1b and conventional B cells. Sem. Immunol. **8**: 29-35.

Kantor A.B., and Herzenberg L.A. (1993). Origin of murine B cell lineages. Ann. Rev. Immunol. **11**: 501-538.

Kroese F.G.M., Butcher E.C., Stall A.M., Lalor P.A., Adams S., and Herzenberg L.A. (1989). Many of the IgA producing plasma cells in murine gut are derived from self-replenishing precursors in the peritoneal cavity. Int. Immunol. **1**: 75-84.

Kroese F.G.M., Cebra J.J., Cammen M.J.F. v.d., Kantor A.B., and Bos N.A. (1995). Contribution of B1 cells to intestinal IgA production in the mouse. Methods, A Companion to Methods in Enzymology **8**: 37-43.

Kroese F.G.M., De Waard R., and Bos N.A. (1996). B1 cells and their reactivity with the murine intestinal microflora. Sem. Immunol. **8**: 11-18.

Lebman D.A., and Coffman R.L. (1988). The effects of IL-4 and Il-5 on the IgA response by murine Peyer's patches B cell subpopulations. J. Immunol. **141**: 2050-2056.

Lebman D.A., Nomura D.Y., Coffman R.L., and Lee F.L. (1990). Molecular characterization of germline immunoglobulin A

transcript produced during transforming growth factor type $\beta$-induced isotype switching. Proc. Natl. Acad. Sci. USA **87**: 3962-3966.

Lin Y.C., and Stavnezer J. (1992). Regulation of transcription of the germline Ig alpha constant region gene by an ZTF element and by novel transforming growth factor beta-1 responsive elements. J. Immunol. **149**: 2914-2925.

Orlandi R., Güssow D.H., Jones P.T., and Winter G. (1989). Cloning immunoglobulin variable domains for expression by the polymerase chain reaction. Proc. Natl. Acad. Sci. USA **86**: 3833-3837.

Stall A.M., Well S.M., and Lam K.-P. (1996). B-1 cells: Unique origins and functions. Sem. Immunol. **8**: 45-59.

Tseng J. (1984). A population of resting IgM-IgD double bearing lymphocytes in Peyer's patches: The major precursor cells for IgA plasma cells in the gut lamina propria. J. Immunol. **132**: 2730-2735.

Van der Heijden P.J., Stok W., and Bianchi A.T.J. (1987). Contribution of immunoglobulin-secreting cells in the murine small intestine to the "background" immunoglobulin production. Immunology **62**: 551-555.

Weinstein P.D., Schweitzer P.A., Cebra-Thomas J.A., and Cebra J.J. (1991). Molecular genetic features reflecting the preference for isotype switching to IgA expression by Peyer's patch germinal center B cells. Int. Immunol. **3**: 1253-1263.

Whitmore A.C., Prowse D.M., Haughton G., and Arnold LW. (1991). Ig isotype switching in B lymphocytes. The effect of T cell-derived interleukins, cytokines, cholera toxin, and antigen on isotype switch frequency of a cloned B cell lymphoma. Int. Immunol. **3**: 95-103.

